# Natural Killer Cell Presence in Antibody-Mediated Rejection

**DOI:** 10.3389/ti.2024.13209

**Published:** 2024-06-24

**Authors:** Matthias Diebold, Evan A. Farkash, Jenna Barnes, Heinz Regele, Nicolas Kozakowski, Martina Schatzl, Katharina A. Mayer, Susanne Haindl, Hannes Vietzen, Luis G. Hidalgo, Philip F. Halloran, Farsad Eskandary, Georg A. Böhmig

**Affiliations:** ^1^ Division of Nephrology and Dialysis, Department of Medicine III, Medical University of Vienna, Vienna, Austria; ^2^ Clinic for Transplantation Immunology and Nephrology, University Hospital Basel, Basel, Switzerland; ^3^ Department of Pathology, University of Michigan, Ann Arbor, MI, United States; ^4^ Department of Pathology, Medical University of Vienna, Vienna, Austria; ^5^ Center for Virology, Medical University of Vienna, Vienna, Austria; ^6^ Department of Surgery, University of Wisconsin, Madison, WI, United States; ^7^ Alberta Transplant Applied Genomics Centre, Faculty of Medicine and Dentistry, University of Alberta, Edmonton, AB, Canada

**Keywords:** natural killer cell, antibody-mediated rejection, microvascular inflammation, immunohistochemistry, genetics

## Abstract

Transcript analyses highlight an important contribution of natural killer (NK) cells to microvascular inflammation (MVI) in antibody-mediated rejection (ABMR), but only few immunohistologic studies have quantified their spatial distribution within graft tissue. This study included 86 kidney transplant recipients who underwent allograft biopsies for a positive donor-specific antibody (DSA) result. NK cells were visualized and quantified within glomeruli and peritubular capillaries (PTC), using immunohistochemistry for CD34 alongside CD16/T-bet double-staining. Staining results were analyzed in relation to histomorphology, microarray analysis utilizing the Molecular Microscope Diagnostic System, functional NK cell genetics, and clinical outcomes. The number of NK cells in glomeruli per mm^2^ glomerular area (NK_glom_) and PTC per mm^2^ cortical area (NK_PTC_) was substantially higher in biopsies with ABMR compared to those without rejection, and correlated with MVI scores (NK_glom_ Spearman’s correlation coefficient [SCC] = 0.55, *p* < 0.001, NK_PTC_ 0.69, *p* < 0.001). In parallel, NK cell counts correlated with molecular classifiers reflecting ABMR activity (ABMR_prob_: NK_glom_ 0.59, NK_PTC_ 0.75) and showed a trend towards higher levels in association with high functional *FCGR3A* and *KLRC2* gene variants. Only NK_PTC_ showed a marginally significant association with allograft function and survival. Our immunohistochemical results support the abundance of NK cells in DSA-positive ABMR.

## Graphical Abstract

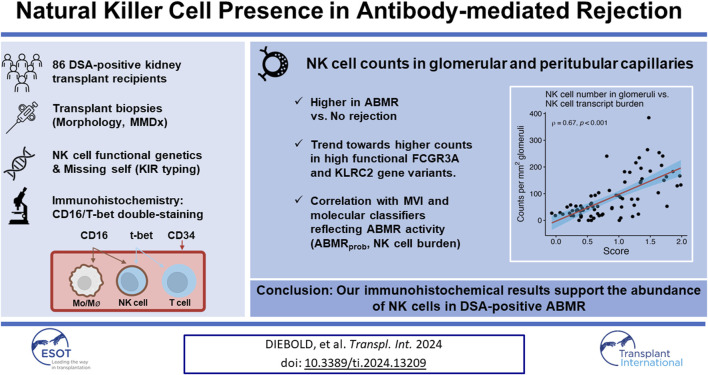

## Introduction

Antibody-mediated rejection (ABMR) is a leading cause of graft failure after kidney transplantation [1]. The current paradigm of the pathogenetic sequence underlying ABMR may involve alloantigen-driven B cell activation and subsequent differentiation into plasma cells producing donor-specific antibodies (DSA) [2, 3]. DSA directed against donor HLA molecules bind to the respective alloantigen expressed on the endothelial surface and may initiate inflammation/injury in the microvasculature, through direct cellular effects, complement activation and/or Fc receptor-mediated activation of innate immune cells, in particular, natural killer (NK) cells and macrophages [4–7]. However, this traditional view may not fully account for the diversity of rejection phenotypes [8]. For instance, a significant subset of patients presenting with microvascular inflammation (MVI), the hallmark lesion of ABMR, may not exhibit detectable antibodies [8, 9]. MVI, DSA negative and C4d negative, may be due to high affinity DSA absorbed to the allograft, or, alternatively, could encompass rejection triggered by “missing self” or other genetically determined mechanisms of NK cell activation [8, 10, 11]. Notwithstanding, in MVI, the role of NK cells as effector cells remains paramount, regardless of the initial trigger of immune activation [4, 8, 12–14]. NK cells are commonly defined as CD3^−^CD56^+^NKp46^+^, and are further categorized into a variety of subsets, including CD56^bright^ and CD56^dim^ cells [15]. Among these, CD56^dim^ cells, particularly those expressing high levels of CD16, may be the predominant subset involved in ABMR [16, 17].

The current assumption of a critical involvement of NK cells in ABMR is primarily based on the results of molecular studies, including bulk and spatial transcriptomics or single cell sequencing [4, 12–14, 18]. However, immunohistologic studies to visualize the actual extent and compartmental distribution of NK cell infiltrates in rejecting allografts are scarce. Available studies mostly relied on single antigen staining (CD56, CD16, or NKp46), which may impede the unambiguous identification of NK cells [6, 19, 20].

The goal of the present study was to provide a detailed morphologic analysis of the presence of capillary NK cells in a well-characterized cohort of 86 DSA-positive renal allograft recipients using immunohistochemical double staining. To gain a more comprehensive understanding of NK cell-associated injury, immunohistologic results were evaluated in relation to genetic determinants of NK cell functionality, the results of detailed morphologic and molecular biopsy analysis, as well as clinical outcome parameters, including graft function and survival.

## Materials and Methods

### Study Design and Patients

The present study comprised 86 DSA-positive kidney transplant recipients who had been recruited during the cross-sectional screening phase (between October 2013 and February 2015) of a single-center randomized controlled trial assessing the effect of bortezomib in late ABMR (BORTEJECT; ClinicalTrials.gov: NCT01873157) [21]. ABMR screening involved 741 adult recipients with stable allograft function at least 180 days post-transplantation (estimated glomerular filtration rate [eGFR] >20 mL/min per 1.73 m^2^). Among 111 DSA-positive recipients 86 underwent renal allograft biopsies [21]. The study was approved by the institutional review board of the Medical University of Vienna and adhered to the principles of the Declaration of Helsinki 2008 and the Declaration of Istanbul. We followed the STROBE guidelines for reporting the study results [22].

### Transplant Biopsies

#### Morphologic Evaluation

As outlined previously [21], we used formalin-fixed and paraffin-embedded tissue sections for histomorphologic and immunohistochemical evaluation. Eighty-four of the 86 study biopsies underwent additional ultrastructural evaluation. The biopsies were analyzed by two experienced renal transplant pathologists (N.K., H.R.). Following the rules of the 2019 Banff schema [23], rejection phenotypes were categorized according to a combination of histologic, immunohistochemical (C4d), ultrastructural and molecular (molecular ABMR classifier) criteria. MVI was quantified using a sum score of glomerulitis (g) and peritubular capillaritis (ptc). Notably, g or ptc scores could not be ascertained for 5 biopsies, limiting the calculation of MVI scores to 81 biopsies.

**FIGURE 1:**
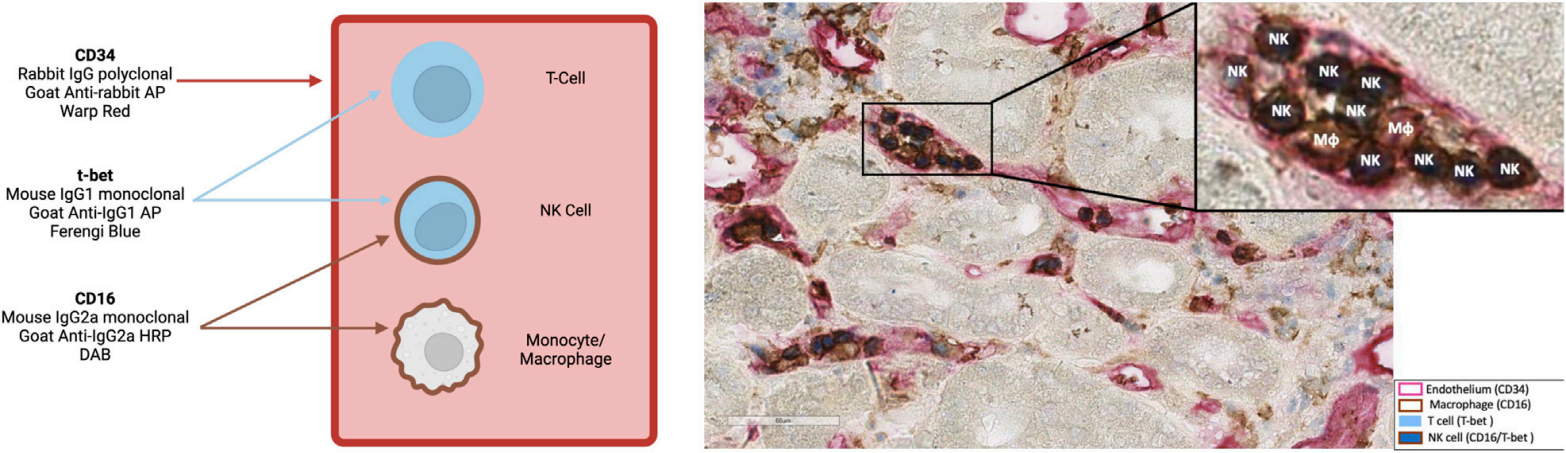
A representative biopsy at 400x showing natural killer (NK) cells within glomerular and peritubular capillaries („NK”). NK cells were identified through a dual CD16 (membrane, brown) and T-bet (nuclear, blue) staining. Endothelial cells are highlighted via CD34 staining (red). MΦ represent macrophages. Representation was created with BioRender.com.

The number and distribution of glomerular and peritubular capillary NK cells were visualized applying T-bet (T cells; NK cells) and CD16 (NK cells; monocytes/macrophages) double-staining as well as CD34 staining to highlight layers of endothelial cells (Figure 1) [24]. For immunohistochemistry, we used polyclonal rabbit CD34-specifc IgG (1:100/Thermo Fisher Scientific, Waltham, Massachusetts, United States) and an alkaline phosphatase (AP)-labeled goat anti-rabbit IgG polyclonal antibody (1:100/Southern Biotech, Birmingham, Alabama, United States), a mouse anti-T-bet IgG1 monoclonal antibody (1:50/4B10/BioLegend, San Diego, California, United States) and an AP-labeled goat anti-mouse IgG1 polyclonal antibody (1:50/Southern Biotech, Birmingham, Alabama, United States), as well as a mouse CD16-specific IgG2a monoclonal antibody (1:20/2H7/GeneTex, Irvine, California, United States) and a horse radish peroxidase-labeled goat anti-mouse IgG2a polyclonal antibody (1:50/Southern Biotech, Birmingham, Alabama, United States). Stained slides were scanned utilizing an Aperio AT2 scanner and visualized using Aperio ImageScope software (Leica, Wetzlar, Hesse, Germany). NK cell staining results were analyzed independently by two experts blinded to the histologic results. Dual-stained cells in glomeruli were manually quantified, and the glomeruli were counted and manually outlined to quantify the glomerular area using ImageScope (Leica, Wetzlar, Hesse, Germany). The same approach was used to quantify dual-stained cells in the peritubular capillaries (PTC) and to calculate the cortical area. The median of the two calculations was divided by the area of the glomeruli for NK cells in the glomeruli or by the area of the cortex (excluding the area of the glomeruli) for NK cells in the PTC. For two patients (2.3%), biopsy specimens were not adequate for evaluation of NK cells in glomeruli. Figure 1 depicts representative examples of immunohistological NK cell staining. There was a strong agreement between the measurements of the two evaluators (Supplementary Figure S1).

#### Molecular Analysis

Eighty-three of the 86 study biopsies were subjected to gene expression profiling using the Molecular Microscope Diagnostic System (MMDx) [25]. Employing a reference dataset of 1,679 indication biopsies, specific classifiers indicative of rejection (ABMR, “all rejection”) and pathogenesis-associated transcript sets were generated as previously detailed [25].

### HLA Antibody Detection

DSA were detected and defined as described previously [21]. Briefly, for HLA antibody detection, LABscreen single-antigen flow-bead assays (One Lambda, Canoga Park, CA) were used. Serum specimens were pretreated with heat inactivation for 30 min at 56°C to mitigate complement-mediated interference. Donor specificity of antibody patterns was analyzed in relation to low- or high-resolution HLA typing for HLA-A, HLA-B, HLA-Cw, HLA-DR, HLA-DQ, and/or HLA-DP. Assay results were reported as mean fluorescence intensity (MFI) of the immunodominant DSA, with an MFI value above 1,000 defined as a positive result.

### KIR Typing for Missing Self Calculation and Functional Single Gene Variants

The inhibitory killer cell Ig-like receptors (KIRs) receptors 2DL1, 2DL2, 2DL3, 3DL1, and 3DL2 were genotyped using the Olerup SSP KIR Genotyping Kit (CareDx Inc., Brisbane, CA, United States), with DNA amplified via PCR-SSP and analyzed through electrophoresis on 2% E-Gel™ Agarose Gels as detailed previously [11]. Missing self was defined based on the absence of specific HLA class I molecules corresponding to the educated NK cells’ KIR receptors [10, 11]. Due to the absence of donor HLA C typing in records before 2009 and unavailable biobanked donor DNA, missing self could only be assessed for 80 donor/recipient pairs.

All patients were genotyped for polymorphisms in four different single genes known to determine the functionality and phenotypic distribution of NK cells as previously detailed [11]. *FCGR3A* ^V/F158^ functional variants (rs396991) determining the affinity of Fc gamma receptor IIIA (FcγRIIIA) were genotyped utilizing the QuantStudio5 real-time polymerase chain reaction (RT-PCR) system (Applied Biosystems, Darmstadt, Germany) alongside the TaqMan SNP Genotyping Assay and TaqMan Genotyping Master Mix [5]. Additionally, *KLRC2* ^wt/del^ gene variants, which encode the NKG2C receptor, were genotyped through touchdown techniques [26]. *KLRK1* ^LNK/HNK^ variants (rs1049174) associated with NKG2D activity were identified using the TaqMan SNP Genotyping Assay and TaqMan Universal PCR Master Mix (Thermo Fisher Scientific, Waltham, MA, United States). Lastly, the functionally relevant rs9916629-C/T polymorphism was examined via an in-house TaqMan assay [27].

### Statistics

Categorical data are reported as frequencies (percentages) and continuous data as median and interquartile range (IQR), respectively. Group comparisons were conducted using the Mann-Whitney-U-Test for continuous variables and Pearson’s chi-square test for categorical variables. Hypothesis testing was two-tailed, with a *p*-value of less than 0.05 indicating statistical significance. Associations between continuous variables were analyzed using Spearman’s rank correlation. For analysis of time-to-event outcomes and calculation of the eGFR slopes, the total number of NK cells was stratified into values above and below the median. Death-censored graft survival was evaluated using Kaplan-Meier method, with group differences assessed using the log-rank test. The Chronic Kidney Disease Epidemiology Collaboration (CKD-EPI) eGFR slope was determined using a linear mixed-effects model. This model incorporated time, the number of NK cells as well as the interaction between the number of NK cells and time, as fixed effects, and random intercepts and slopes through an unstructured covariance matrix. Statistical analyses were performed using R software, version R 4.0.2 (R Core Team 2020. R: a language and environment for statistical computing. R Foundation for Statistical Computing,^1^). A list of the packages used for this analysis is provided as Supplementary Material.

## Results

### Patient Characteristics

The study cohort consisted of 86 renal allograft recipients, all of whom underwent allograft biopsies for a positive DSA screening result. Fifty patients were diagnosed with ABMR according to the Banff schema (active ABMR: n = 15; chronic active ABMR: n = 33; chronic [inactive] ABMR: n = 2), and the median MVI score was 2 (IQR 0–3). Baseline characteristics are provided in Table 1. The median recipient age was 47 years (IQR 36–54) and 39 (45.3%) patients were female. Fourteen (16.3%) patients were recipients of a living donor transplant, 25 (29.1%) recipients of a re-transplant, and 26 (30.2%) patients were pre-sensitized and were subjected to desensitization with immunoadsorption and depleting antibody induction at the time of transplantation. The median eGFR at the time of ABMR screening was 54 mL/min/1.73 m^2^ (IQR 32–78), with most recipients being on tacrolimus-based maintenance therapy.

**TABLE 1:** Baseline characteristics.

Parameter	No ABMR (n = 36)	ABMR (n = 50)	Total (N = 86)	*p*-value
**Variables recorded at transplantation**				
Recipient age (yr), median (IQR)	47 (39–54)	48 (35–54)	47 (36–54)	0.575
Female recipient sex, no. (%)	14 (38.9)	25 (50.0)	39 (45.3)	0.423
Donor age^a^ (yr), median (IQR)	44 (36–54)	46 (33–58)	46 (36–58)	0.757
Prior kidney transplant, no. (%)	10 (27.8)	15 (30.0)	25 (29.1)	1.000
Living donor, no. (%)	6 (16.7)	8 (16.0)	14 (16.3)	1.000
ABO-incompatible live donor transplant^b^, n (%)	1 (2.8)	0 (0.0)	1 (1.2)	0.868
Cold ischemia time^a^ (hr), median (IQR)	11 (5–15)	12 (9–18)	12 (9–17)	0.187
HLA mismatch^a^ (A, B, DR), median (IQR)	3 (3–4)	3 (2–3)	3 (2–4)	0.053
CDC panel reactivity^a^ ≥10%, no. (%)	6 (16.7)	9 (18.0)	15 (17.4)	1.000
Preformed anti-HLA DSA^c^, no. (%)	5 (13.9)	20 (40.0)	25 (29.1)	0.009
Induction with antithymocyte globulin, n (%)	6 (16.7)	22 (44.0)	28 (32.6)	0.015
Peritransplant immunoadsorption^d^, n (%)	6 (16.7)	20 (40.0)	26 (30.2)	0.037
**Variables recorded at the time of ABMR screening**				
Time to biopsy (yr), median (IQR)	5 (2–12)	5 (2–13)	5 (2–13)	0.793
Recipient age (yr), median (IQR)	55 (48–63)	55 (43–61)	55 (45–62)	0.581
MFI of peak DSA	1,491 (1,205 to 3,446)	3,878 (2,223 to 10,484)	2,952 (1,485 to 6,781)	<0.001
eGFR (ml/min/1.73 m^2^), median (IQR)	58 (40–82)	44 (30–76)	54 (32–78)	0.183
Urinary protein/creatinine ratio (mg/g), median (IQR)	167 (73–270)	258 (86–954)	192 (80–421)	0.052
Triple immunosuppression, no. (%)	27 (75.0)	38 (76.0)	65 (75.6)	1.000
Tacrolimus-based immunosuppression, no. (%)	21 (58.3)	31 (62.0)	52 (60.5)	0.905
*Index biopsy results*				
Active ABMR, no. (%)	0 (0.0)	15 (30.0)	15 (17.4)	0.001
Chronic active ABMR, no. (%)	0 (0.0)	33 (66.0)	33 (38.4)	<0.001
Chronic inactive ABMR, no. (%)	0 (0.0)	2 (4.0)	2 (2.3)	0.625
Microvascular inflammation (MVI)^e^, median (IQR)	0 (0–0)	2 (2–4)	2 (0–3)	<0.001
C4d positivity, no. (%)	2 (5.6)	24 (48.0)	26 (30.2)	<0.001
Banff single lesion scores, median (IQR)				
Glomerulitis (g)	0 (0–0)	1 (1–2)	0 (0–2)	<0.001
Peritubular capillaritis (ptc)	0 (0–0)	2 (1–2)	0 (0–2)	<0.001
Interstitial inflammation (i)	0 (0–0)	0 (0–0)	0 (0–0)	0.704
Tubulitis (t)	0 (0–0)	0 (0–0)	0 (0–0)	0.341
Glomerular basement membrane double contours (cg)	0 (0–0)	1 (0–1)	0 (0–1)	<0.001
Interstitial fibrosis (ci)	1 (0–1)	1 (1–1)	1 (1–1)	0.028
Tubular atrophy (ct)	1 (0–1)	1 (1–1)	1 (0–1)	0.049
Total inflammation (ti)	1 (0–1)	1 (0–1)	1 (0–1)	0.735
Vascular fibrous intimal thickening (cv)^f^	1 (0–1)	1 (0–1)	1 (0–1)	0.854
Interstitial fibrosis and tubular atrophy (IFTA)	2 (0–4)	3 (2–4)	3 (1–4)	0.093

ABMR, antibody-mediated rejection; CDC, complement-dependent cytotoxicity; DSA, donor-specific antibody; eGFR, estimated glomerular filtration Rate; HLA, human leukocyte antigen; IQR, interquartile range; MFI, mean fluorescence intensity.

^a^ Donor age, cold ischemia time, HLA, mismatch and CDC, panel reactivity were not recorded for 3, 5, 1, and 5 recipients, respectively.

^b^ This patient underwent desensitization for ABO (AB, donor to O recipient) plus HLA, antibody (DSA+) barriers.

^c^ Pretransplant single-antigen testing was available for 42 patients (solid-phase HLA, antibody screening on the wait list according to our local standard implemented in July 2009).

^d^ Pre-sensitized patients (until 2009: ≥40% CDC, panel reactivity; since 2009: preformed DSA) were subjected to a protocol of peritransplant immunoadsorption as earlier detailed [35].

^e^ g and/or ptc single lesions scores could not be ascertained for 5 biopsies, limiting the calculation of MVI, scores to 81 biopsies.

^f^ The vascular fibrous intimal thickening score was not documented for two recipients in the bortezomib group and five recipients in the placebo group due to inadequate biopsy material for complete lesion scoring.

### NK Cell Presence and ABMR Histomorphology

The number of glomeruli, the glomerular and the cortical area calculated for each sample were comparable between biopsies with and without ABMR (Table 2). The median sum of NK cells in glomerular capillaries was significantly higher in patients with ABMR compared to those without ABMR (NK cell number per mm^2^ glomerular area: 103, IQR 47 to 180 versus 36, IQR 18 to 49; *p* < 0.001). NK cell counts in glomeruli were numerically highest in active ABMR, and lowest in chronic ABMR (Supplementary Table S1). The median number of NK cells in PTC per mm^2^ cortical area was also higher in patients with ABMR (24, IQR 11 to 33 versus 3 IQR 2 to 5; *p* < 0.001), particularly in active and chronic active ABMR (Supplementary Table S1).

**TABLE 2:** Intracapillary NK cell counts in relation to ABMR diagnosis.

Variables	No ABMR (n = 36)	ABMR (n = 50)	Total (N = 86)	*p*-value
Cortical area, mm^2^ (IQR)	5 (3–7)	4 (3–6)	4 (3–7)	0.353
Glomerular area, mm^2^ (IQR)	0.16 (0.11–0.31)	0.15 (0.08–0.26)	0.15 (0.10–0.26)	0.255
Number of glomeruli, median (IQR)	7 (4–12)	6 (4–10)	7 (4–10)	0.231
NK cells in glomeruli^a^ per mm^2^ glomerular area, median (IQR)	36 (18–49)	103 (47–180)	52 (25–130)	<0.001
NK cells in PTC per mm^2^ cortical area, median (IQR)	3 (2–5)	24 (11–33)	10 (3–27)	<0.001

ABMR, antibody-mediated rejection; NK cell, natural killer cell.

^a^ Sufficient material for calculation of glomerular and total NK, cell counts was available for 84/86 patients.

**FIGURE 2:**
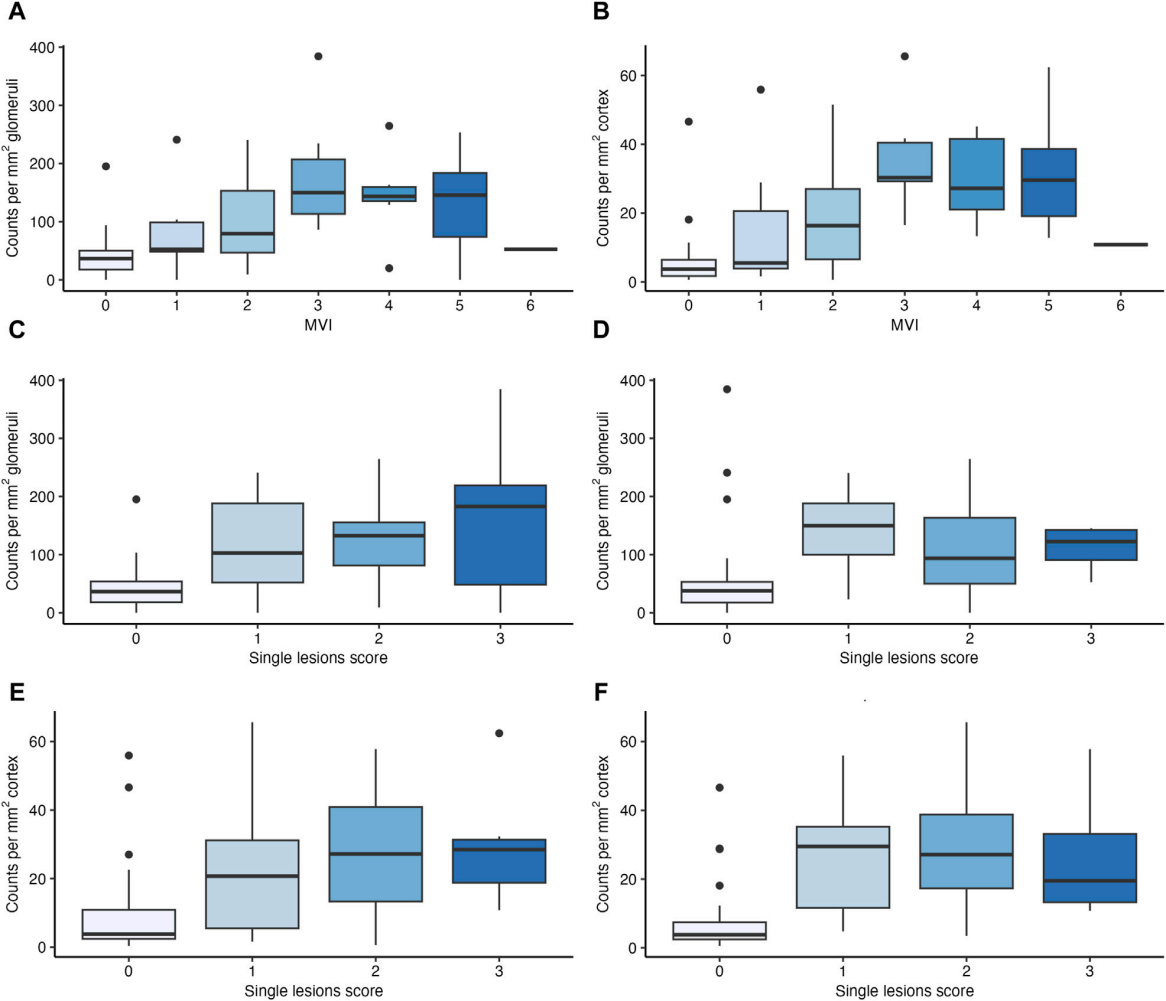
Number of natural killer (NK) cells in relation to features of microvascular inflammation (MVI). Boxplots depicting the number of NK cells in glomeruli and peritubular capillaries (PTC) in relation to MVI scores **(A,B)**, to glomerulitis (g) scores **(C,E)** and peritubular capillaritis (ptc) **(D,F)** scores.

As shown in Figure 2, Supplementary Table S2 and Supplementary Figure S2, glomerular NK cell counts were tightly correlated with MVI scores (Spearman’s correlation coefficient [SCC] = 0.55, *p* < 0.001) as well as g (SCC = 0.51, *p* < 0.001) and ptc (SCC = 0.48, *p* < 0.001) Banff single lesion scores. Stronger correlations were found between peritubular capillary NK cell counts and MVI scores (SCC = 0.69, *p* < 0.001) as well as g (SCC = 0.54, *p* < 0.001) and ptc (SCC = 0.69, *p* < 0.001) Banff single lesion scores. No such correlations were observed for other single lesions, including tubulitis (t), interstitial infiltrates (i) or lesions reflecting chronic injury in the tubulo-interstitium (ci, ct), arteries (cv) or glomeruli (cg). No significant correlation was found between immunodominant DSA MFI and the number of NK cells in glomeruli (SCC = 0.21, *p* = 0.058) but we observed a weak correlation with NK cell counts in PTC (SCC = 0.32, *p* = 0.003). We did not find any associations with other clinical (eGFR, protein/creatinine ratio) or immunological characteristics (HLA mismatch) (data not shown).

### NK Cell Presence and Gene Expression Patterns

#### NK Cells in Glomeruli

**FIGURE 3:**
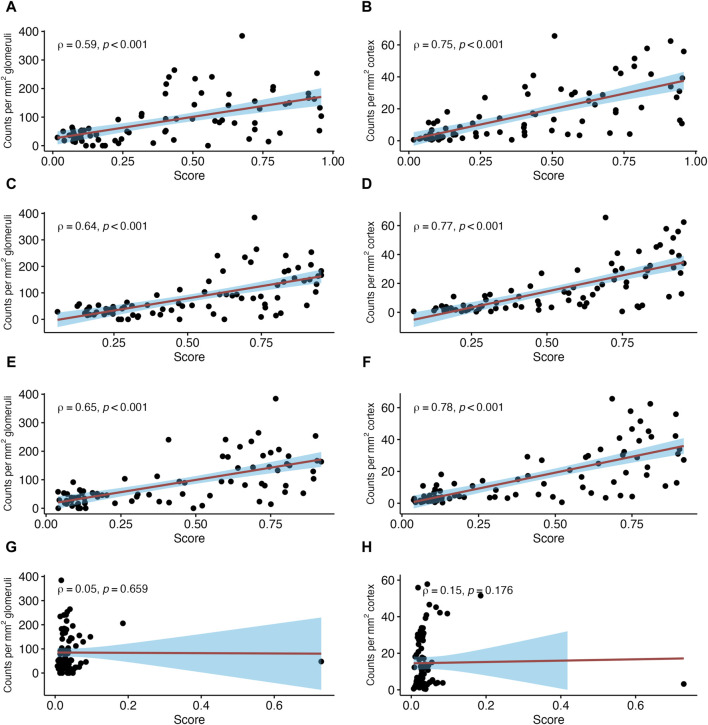
Number of natural killer (NK) cells in relation to antibody-mediated rejection (ABMR)-related molecular classifier scores. Shown are correlations between NK cell counts in glomeruli and peritubular capillaries (PTC) per mm2 glomerular/cortical area and Molecular Microscope Diagnostic System (MMDx)-derived scores reflecting antibody-mediated rejection. ABMR activity (ABMR_prob_) **(A,B)**, probability of lesion scores for glomerulitis >0 (g_prob_) **(C,D)**, probability of peritubular capillaritis >0 (ptc_prob_) **(E,F)** and TCMR classifier (TCMR_prob_) **(G,H)**. Red lines indicate linear regression with the 95% confidence interval shown in blue. ρ denotes the Spearman’s correlation coefficient.

Next, we investigated associations between NK cell counts in glomeruli per mm^2^ glomerular area and distinct MMDx-derived molecular scores (Figure 3). Strong correlations were found with molecular classifiers that reflect the probability of histologic ABMR (ABMR_prob_; SCC = 0.59, *p* < 0.001), or the probability of a g score >0 (g_prob_; SCC = 0.64, *p* < 0.001) or a ptc score >0 (ptc_prob_; SCC = 0.65, *p* < 0.001). Conversely, no significant correlations were observed with the molecular classifier for TCMR diagnosis probability (TCMR_prob_; SCC = 0.05, *p* = 0.659) even after excluding an outlier (SCC = 0.14, *p* = 0.202).

**FIGURE 4:**
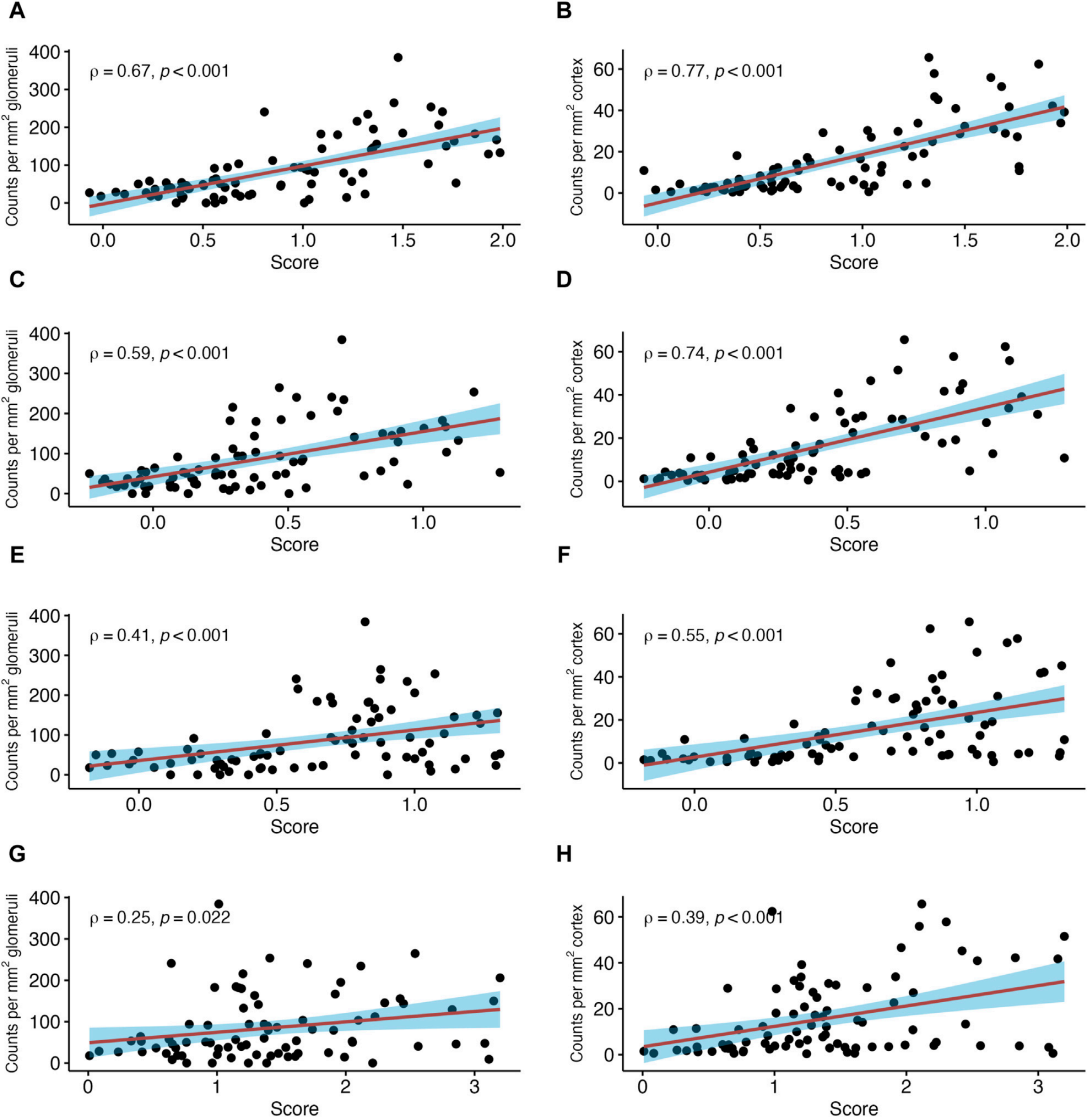
Number of natural killer (NK) cells in relation to selected pathogenesis-based transcript sets (PBT). Shown are correlations between NK cell counts in glomeruli and peritubular capillaries (PTC) per mm2 glomerular/cortical area, respectively, and NK cell transcript burden **(A,B)**, donor-specific antibody (DSA) selective **(C,D)**, gamma inducible (GRIT) **(E,F)** and T cell burden (TCB) transcript sets **(G,H)**. The red line indicates a linear regression line with the 95% confidence interval shown in blue. ρ denotes the Spearman’s correlation coefficient.

Among analyzed pathogenesis-based transcripts (PBT) sets, the strongest correlation was found for a PBT set reflecting the NK cell transcript burden (NKB; SCC = 0.67, *p* < 0.001) (Figure 4). Weaker correlations were observed for a DSA selective (DSAST; SCC = 0.59, *p* < 0.001) and interferon gamma-inducible PBT (GRIT, SCC = 0.49, *p* < 0.001). The correlation with the T cell burden (TCB) was only moderate (SCC = 0.25, *p* = 0.022). The correlations with all rejection’ (Rej_prob_; SCC = 0.63, *p* < 0.001), cg score >0 (cg_prob_; SCC = 0.42, *p* < 0.001) and injury-repair response associated PBT (IRRAT; SCC = 0.15, *p* = 0.145) are shown in Supplementary Figure S3.

#### NK Cells in Peritubular Capillaries

In PTC, stronger correlations were found between NK cells per mm^2^ cortical area and the three ABMR-related classifiers: ABMR_prob_ (SCC = 0.75, *p* < 0.001), g_prob_ (SCC = 0.77, *p* < 0.001) and ptc_prob_ (SCC = 0.78, *p* < 0.001) (Figure 3). The PBT sets related to ABMR also showed strong correlations, with the strongest correlation for NKB (SCC = 0.77, *p* < 0.001). DSAST (SCC = 0.74, *p* < 0.001) and GRIT (SCC = 0.55, *p* < 0.001) showed strong correlations as well (Figure 4). TCB showed only a weak but significant correlation (SCC = 0.39, *p* < 0.001). A weak correlation was also noted with IRRAT (SCC = 0.34, *p* < 0.001). Supplementary Figure S3 shows the correlations with “all rejection” probability (Rej_prob_; SCC = 0.76, *p* < 0.001) and cg score >0 (cg_prob_; SCC = 0.55, *p* < 0.001).

#### Molecular Archetypes Clusters

Investigating molecular rejection archetype clusters, the number of NK cells in glomeruli and in PTC was highest in early-stage ABMR (EABMR), followed by fully developed ABMR (FABMR), late-stage ABMR (LABMR) and no rejection as well as TCMR (both *p* < 0.001) (Supplementary Table S3).

### NK Cell Presence and Functional NK Cell Genetics

Next, we analyzed the association between immunohistochemical results and a set of functional NK cell gene polymorphisms, known to influence the number and functionality of NK cells, as well as missing self. Supplementary Table S3 shows the median number of NK cells stratified by the degree of “missing self,” calculated on the basis of KIR receptor and HLA polymorphisms, as well as individual functional NK cell gene variants, such as *FCGR3A* ^V/F158^, *KLRC2* ^wt/del^, *KLRK1* ^LNK/HNK^ and rs9916629-C/T.

The number of NK cell counts in glomeruli and PTC turned out to be numerically higher in patients with high functional variants *KLRC2* ^wt/wt^ and *FCGR3A* ^V/F158^, without reaching the conventional boundaries for statistical significance (Supplementary Table S4). Only NK cell counts in PTC among patients with *KLRC2* ^wt/wt^ were significantly higher. For the other variants, including missing self, no such associations were observed.

### Number of NK Cells and Transplant Outcomes

**FIGURE 5:**
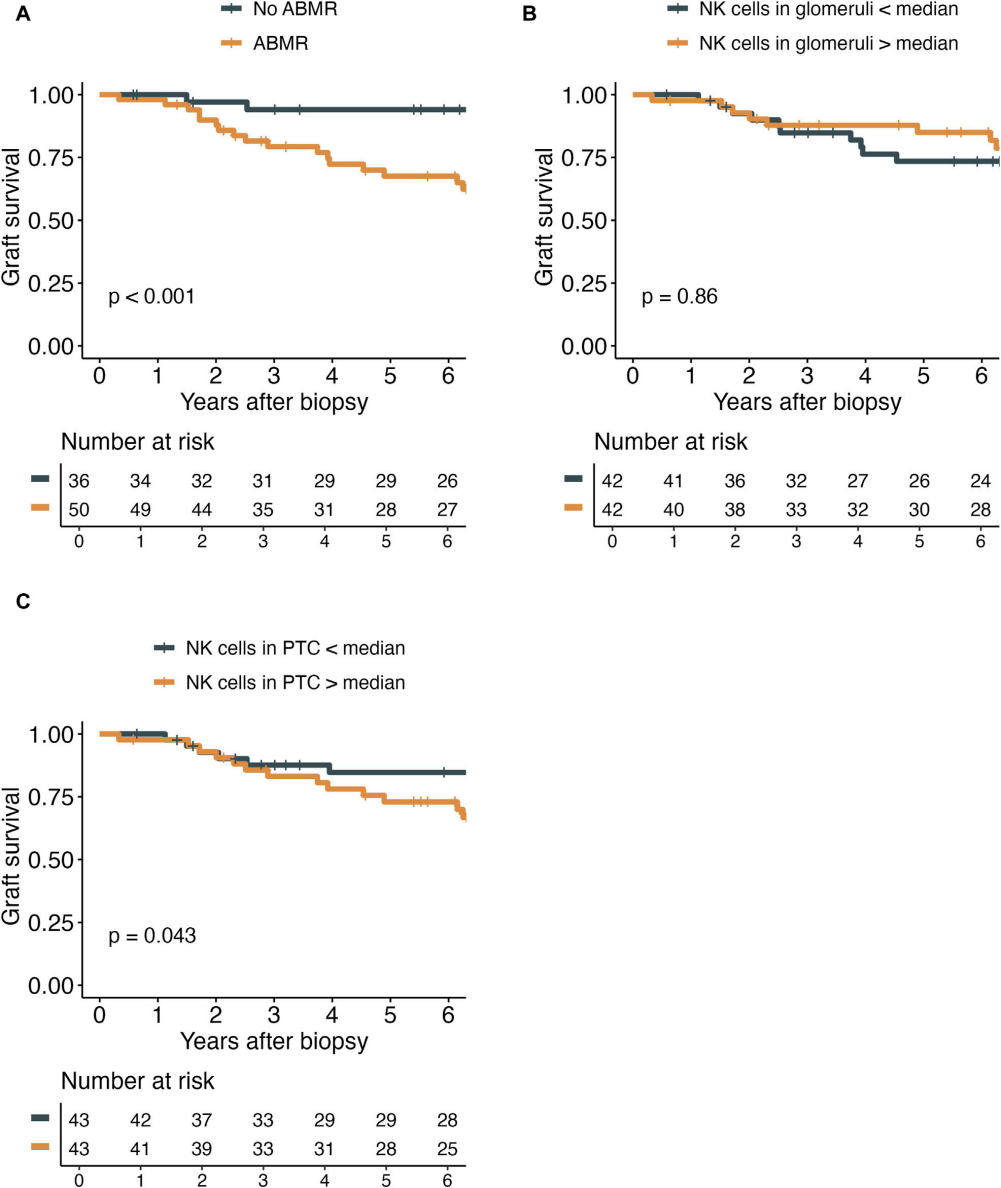
Kaplan-Meier plots demonstrating the death-censored graft survival in relation to antibody-mediated rejection (ABMR) diagnosis **(A)**, the number of NK cells in glomeruli (g) per mm^2^ of glomerular area **(B)**, and the number of NK cells in peritubular capillaries (PTC) per mm^2^ cortical area **(C)**.

The diagnosis of ABMR was significantly associated with inferior death-censored graft survival (Figure 5). Interestingly, the number of NK cells in PTC, but not glomeruli, did have a marginally significant impact on graft survival (*p* = 0.043). Nevertheless, neither the number of NK cells in glomeruli nor the number of NK cells in PTC did impact the yearly eGFR decline (Supplementary Table S5).

## Discussion

A major finding of the present study was that, among a subset of patients diagnosed with ABMR, there was a marked elevation of NK cell counts in glomerular and peritubular capillaries. This increase in NK cell counts was strongly correlated with distinct ABMR-related Banff single lesion scores and the extent of MVI, but not TCMR-related Banff single lesion scores. Additionally, we found a significant correlation between the presence of NK cells and molecular classifiers and transcript sets related to ABMR activity, NK cell burden or DSA effects, while no or only weak correlations were observed with classifiers related to TCMR. Notably, our results extended to functional polymorphisms in *KLRC2* and *FCGR3A* genes, which regulate the abundance and function of NKG2C^+^ NK cells and determine the binding affinity of the FcγRIIIA receptor, respectively. However, despite associations with features of rejection, only a marginal association was found between the number of NK cells in PTC and inferior graft survival.

Several studies have underscored the pivotal role of NK cells in ABMR, primarily inferred from elevated NK cell-dependent transcripts in biopsies from patients with ABMR [12, 14, 18]. Nevertheless, there is a paucity of morphological studies visualizing NK cells in allograft biopsies. Existing data largely rely on single-marker staining (CD16, CD56, or NKp46), which does not allow for definitive identification of NK cells [6, 14, 16, 20, 28, 29]. Kildey et al. [16] successfully identified NK cells among lymphocytes from digested biopsy samples using flow cytometry; however, the methodology precluded analyses of spatial distribution. Our study supports and expands upon these findings by employing a double-staining technique for a more precise identification of NK cell infiltrates. The reliability of our staining technique was supported by the strong correlation observed between our immunohistochemical results and a pathogenesis-based transcript set reflecting the NK cell burden. In this regard, our study extends the original work by Hidalgo et al. [12], where only CD56 as a sole marker was used for the histological detection of NK cells.

NK cells may contribute to rejection through various mechanisms, including antibody-dependent cellular cytotoxicity (ADCC) or direct lysis, resulting from increased genetically determined activation or a lack of inhibition triggered by missing self [8, 17]. ADCC, for instance, may involve the binding of FcγRIIIA to the Fc portions of alloantigen-bound antibodies (e.g., HLA or non-HLA). A pivotal role of FcγRIIIA-positive effector cells in rejection was suggested by a recently published study employing innovative technologies to dissect the involvement of distinct cellular components, including single cell sequencing, spatial transcriptomics or multifluorescent staining [18]. Our previous work has shown that a polymorphism affecting the binding affinity of this receptor is associated with the occurrence and extent of MVI [5, 11]. In the present study, we found that the number of NK cells in transplant capillaries is increased in patients with genotypes including the high-affinity *FCGR3A* ^V158^ allele, although not statistically significant. However, we found an association with a deletion polymorphism in the *KLRC2* gene, which not only influences the expression of the NKG2C receptor but also shifts the entire NK subset towards a higher proportion of NKG2C^+^ NK cells. A potential relevance of this polymorphism, as suggested by its association with MVI, was discussed in previous studies [11, 26]. However, a notable difference was primarily observed in the quantity of NK cells within PTC and the absolute difference was relatively small. Investigating the proportion of NK cells in patients with a homozygous deletion of the *KLRC2* gene would have been of interest, but this aspect could not be explored in our study due to the absence of patients with such a genotype. Other functional polymorphisms had no impact on the number of NK cells. Likewise, no association with the presence of missing self was observed. This finding, however, was not unexpected, as missing self in the same cohort was not associated with the occurrence of MVI [11]. As previously discussed [11], it is possible that in a selected population of patients with circulating DSA, other mechanisms of NK cell activation, such as ADCC, may predominate.

Interestingly, we only found a marginal association between NK cells in PTC and inferior death-censored graft survival, but no association for NK cells in glomeruli. This finding aligns with Yazdani et al. [14], who showed a strong independent effect of NK cell infiltrates on graft survival. The difference of measure of effect could be explained by the different patient’s population and the effect of the number of NK cells on graft survival would be higher in a population of kidney transplant recipients not selected by the presence of DSA. However, it is unclear why we did not find an association for NK cells in glomeruli. Due to the limited sample size, we refrained from conducting further subanalyses.

An interesting observation was that NK cells counts were highest in a molecular archetype reflecting early-onset AMR. This was paralleled by higher glomerular capillary NK cell counts in biopsies showing active, as compared to chronic active or chronic ABMR. In this respect, our data may be in some contrast to a study by Shah et al. [30], who used pathway enrichment analysis, single-cell RNA-Seq data, BayesPrism and immunohistochemistry (CD56 staining only) to identify NK cells. The authors found a higher NK cell–mediated cytotoxicity pathway and NK cell fractions in immunohistochemistry in chronic ABMR compared to active ABMR. However, in contrast to this study, where all but one ABMR cases were diagnosed within 8 weeks after transplantation, our present analysis included only cases of late ABMR (≥180 days post-transplantation; median 5 years post-transplantation). This may have pathophysiological implications leading to different results. For instance, it was suggested that the primary mechanism by which DSA mediate early ABMR is complement-dependent cytotoxicity, whereas NK cell-dependent ADCC is more prominent in late ABMR [30].

Our analysis offers several advantages, including the well-characterized patient population which allowed for a comprehensive analysis of immunohistochemical results with a set of different histological and molecular markers related to rejection, as well as genetic polymorphisms that may determine NK cell functionality and distribution. However, it is important to acknowledge some remaining limitations. First, the patient population consists of a selected cohort of DSA-positive transplant recipients. While this facilitated accurate characterization, it may have reduced the generalizability and, due to the low number of patients, also diminished the power to recognize subtle differences. Our in-depth analysis of the BORTEJECT screening cohort allowed us to dissect the abundance of NK cells only in a very specific context, that is, DSA-positive ABMR. Other MVI variants (e.g., MVI, DSA negative and C4d negative) or TCMR and mixed rejection cases were not represented in our cohort, which precluded the evaluation of a potential role of NK cells in a broader sense. Secondly, it is crucial to recognize that correlations observed in the study do not imply causation. Although the role of NK cells in the pathogenesis of microvascular inflammation is increasingly understood and a relevant role seems very likely, this analysis only shows correlations. Further validations are necessary to strengthen the evidence and establish a causal relationship definitively. Specifically, in this analysis we were only able to morphologically describe the number of capillary NK cells. Whether they act as the primary effectors causing tissue injury or whether they are simply bystander cells cannot be deduced from this analysis. Further research with larger sample sizes and functional assays may be warranted to elucidate the specific role of NK cells in the context of allograft rejection. Notably, in a recently published phase 2 trial evaluating the CD38 antibody felzartamab in late active and chronic active ABMR, we demonstrated a marked reduction in MVI scores alongside a reduction in peripheral NK cell counts and donor-derived cell-free DNA release [31]. The results of this trial may provide further evidence that NK cells could play a role as effector cells promoting graft injury, and may be in line with previous experimental studies that have demonstrated that NK cell depletion can ameliorate rejection processes [4, 32]. One concern may be that our approach of immunohistochemical double-staining, which relies on the detection of CD16-and CD56-positive cells, may not reliably detect CD56^bright^CD16^dim^ cells. While this may have led to an underestimation of total NK cell counts, this distinct subset is most likely not contributing to NK cell-dependent ADCC [15]. However, it is important to note that NK cells are able to shed their CD16 receptor upon lysis of target cells to disassemble the NK cell immune synapse, which allows them to target several other cells in a row [33, 34]. Because of this phenomenon, it could be that some effector cells have escaped immunohistochemical detection in our study biopsies.

In conclusion, our study demonstrated an increased number of NK cells in the glomeruli and PTC, among patients with DSA-positive MVI. The quantity of NK cells showed correlations with histological markers of MVI, as well as with ABMR-related molecular classifiers and pathogenesis-based transcript sets. Polymorphisms in the *FCGR3A* and *KLRC2* genes, known to affect NK cell functionality, were found to correlate with the number of NK cells. However, despite these associations, only the number of NK cells within PTC was prognostic for transplant survival in our analysis. Further research, including analyses of unselected transplant cohorts, is needed to clarify the precise role of NK cells in allograft rejection and transplant outcomes.

## Data Availability

The data analyzed in this study is subject to the following licenses/restrictions: The datasets presented in this article are not readily available because public sharing of individual participant data was not included in the informed consent of the BORTEJECT trial. Data can be made available to interested researchers upon reasonable request by mailing to georg.boehmig@meduniwien.ac.at. Requests to access these datasets should be directed to georg.boehmig@meduniwien.ac.at.

## Footnotes

1
https://www.R-project.org
