# Supplementary material for: Natural Killer Cell Presence in Antibody-Mediated Rejection
Source: Transpl Int. 2024 Jun 24;37:13209. doi: 10.3389/ti.2024.13209 (PMC11228143; doi:10.3389/ti.2024.13209)
Supplement: Supplementary file 1 [file DataSheet1.docx]

Natural Killer Cell Presence in Antibody-mediated Rejection

Matthias Diebold, Evan Farkash, Jenna Barnes, Heinz Regele, Nicolas Kozakowski, Martina Schatzl, Katharina A. Mayer, Susanne Haindl, Hannes Vietzen, Luis G. Hidalgo, Philip F. Halloran, Farsad Eskandary, and Georg A. Böhmig

Table of contents

[List of R packages used 2](#_Toc167879489)

[Supplementary Table 1. Number of NK cells according to Banff ABMR subtypes 3](#_Toc167879490)

[Supplementary Table 2. Number of NK cells and Banff single lesions scores. 4](#_Toc167879491)

[Supplementary Table 3. Number of NK cells - glomeruli and peritubular capillaries in relation to the MMDx cluster analysis for molecular archetypes of ABMR. 6](#_Toc167879492)

[Supplementary Table 4. Number of NK cells in relation to missing self and genotypic variants. 7](#_Toc167879493)

[Supplementary Table 5. Estimated eGFR slope in patients stratified to the median number of NK cells in glomeruli and PTC 9](#_Toc167879494)

[Supplementary Figure 1. Bland-Altman plot for the agreement of the histological evaluation of NK cells between the two investigators. 10](#_Toc167879495)

[Supplementary Figure 2. Correlation plot between histological markers and the number of natural killer (NK) cells. 11](#_Toc167879496)

[Supplementary Figure 3. Number of natural killer (NK) cells in glomeruli and peritubular capillaries (PTC) and distinct Molecular Microscope Diagnostic System (MMDx) classifiers. 12](#_Toc167879497)

# List of R packages used

- Tidyverse: Wickham H, Averick M, Bryan J, Chang W, McGowan LD, François R, Grolemund G, Hayes A, Henry L, Hester J, Kuhn M, Pedersen TL, Miller E, Bache SM, Müller K, Ooms J, Robinson D, Seidel DP, Spinu V, Takahashi K, Vaughan D, Wilke C, Woo K, Yutani H (2019). “Welcome to the tidyverse.” _Journal of Open Source Software_, *4*(43), 1686. doi:10.21105/joss.01686 <https://doi.org/10.21105/joss.01686>.
- Finalfit: Harrison E, Drake T, Ots R (2023). _finalfit: Quickly Create Elegant Regression Results Tables and Plots when Modelling_. R package version 1.0.6, <https://CRAN.R-project.org/package=finalfit>.
- Lme4: Douglas Bates, Martin Maechler, Ben Bolker, Steve Walker (2015). Fitting Linear Mixed-Effects Models using lme4. Journal of Statistical Software, 67(1), 1-48. doi:10.18637/jss.v067.i01.
- lmerTest: Kuznetsova A, Brockhoff PB, Christensen RHB (2017). “lmerTest Package: Tests in Linear Mixed Effects Models.” _Journal of Statistical Software_, *82*(13), 1-26. doi:10.18637/jss.v082.i13 <https://doi.org/10.18637/jss.v082.i13>.
- ggplot2: Wickham H (2016). ggplot2: Elegant Graphics for Data Analysis. Springer-Verlag New York. ISBN 978-3-319-24277-4, <https://ggplot2.tidyverse.org>.
- blandr: Datta D (2017). blandr: a Bland-Altman Method Comparison package for R. [doi:10.5281/zenodo.824514](https://doi.org/10.5281/zenodo.824514), <https://github.com/deepankardatta/blandr>.
- ggpubr: Kassambara, A. (2022). Ggpubr: ‘Ggplot2’ Based Publication Ready Plots.
  https://cran.r-project.org/web/packages/ggpubr/index.html

# Supplementary Table 1. Number of NK cells according to Banff ABMR subtypes

|  | Active ABMR | Chronic active ABMR | Chronic inactive ABMR | p |
| --- | --- | --- | --- | --- |
| NK cells in glomeruli per mm^2^ glomerular area | 141.3 (67.9 to 185.6) | 98.6 (46.9 to 180.5) | 11.6 (5.8 to 17.3) | 0.084 |
| NK cells in PTC per mm^2^ cortical area | 22.6 (9.4 to 30.1) | 27.0 (12.2 to 39.2) | 3.0 (2.3 to 3.7) | 0.096 |
| ABMR, antibody-mediated rejection; NK cells, natural killer cells, values are median and IQR | | | | |

# Supplementary Table 2. Number of NK cells and Banff single lesions scores.

| Banff single lesions scores | 0 | 1 | 2 | 3 |
| --- | --- | --- | --- | --- |
| g score |  |  |  |  |
| NK cells in glomeruli per mm^2^ glomerular area | 36 (18 to 54) | 103 (52 to 188) | 133 (81 to 156) | 183 (48 to 219) |
| NK cells in PTC per mm^2^ cortical area | 4 (2 to 11) | 21 (5 to 31) | 27 (13 to 41) | 28 (19 to 31) |
| ptc score |  |  |  |  |
| NK cells in glomeruli per mm^2^ glomerular area | 38 (18 to 53) | 150 (100 to 188) | 94 (50 to 163) | 122 (91 to 142) |
| NK cells in PTC per mm^2^ cortical area | 4 (2 to 7) | 30 (12 to 35) | 27 (17 to 39) | 20 (13 to 33) |
| t score |  |  |  |  |
| NK cells in glomeruli per mm^2^ glomerular area | 51 (26 to 116) | 96 (66 to 151) | 129 (73 to 185) | 48 (48 to 48) |
| NK cells in PTC per mm^2^ cortical area | 10 (4 to 27) | 32 (10 to 43) | 16 (9 to 23) | 3 (3 to 3) |
| i score |  |  |  |  |
| NK cells in glomeruli per mm^2^ glomerular area | 51 (26 to 135) | 90 (39 to 103) | 48 (48 to 48) |  |
| NK cells in PTC per mm^2^ cortical area | 11 (4 to 27) | 9 (3 to 35) | 3 (3 to 3) |  |
| cg score |  |  |  |  |
| NK cells in glomeruli per mm^2^ glomerular area | 49 (26 to 94) | 96 (23 to 176) | 84 (39 to 142) | 73 (51 to 116) |
| NK cells in PTC per mm^2^ cortical area | 5 (3 to 18) | 21 (6 to 32) | 21 (13 to 30) | 14 (11 to 28) |
| ct score |  |  |  |  |
| NK cells in glomeruli per mm^2^ glomerular area | 45 (25 to 150) | 51 (32 to 128) | 94 (54 to 135) | 23 (16 to 24) |
| NK cells in PTC per mm^2^ cortical area | 4 (2 to 25) | 12 (4 to 29) | 17 (6 to 27) | 4 (3 to 5) |
| ci score |  |  |  |  |
| NK cells in glomeruli per mm^2^ glomerular area | 50 (32 to 120) | 49 (24 to 93) | 94 (27 to 138) | 50 (25 to 96) |
| NK cells in PTC per mm^2^ cortical area | 3 (2 to 18) | 12 (6 to 27) | 18 (5 to 40) | 10 (4 to 19) |
| cv score |  |  |  |  |
| NK cells in glomeruli per mm^2^ glomerular area | 50 (26 to 162) | 53 (17 to 110) | 46 (23 to 58) | 95 (42 to 142) |
| NK cells in PTC per mm^2^ cortical area | 6 (2 to 27) | 8 (3 to 32) | 11 (4 to 13) | 19 (11 to 28) |
| NK cells, natural killer cells, PTC, peritubular capillaries.  Values are median and IQR | | | | |

# Supplementary Table 3. Number of NK cells - glomeruli and peritubular capillaries in relation to the MMDx cluster analysis for molecular archetypes of ABMR.

| Variables | EABMR n=13 | FABMR n=23 | LABMR n=4 | NR n=40 | TCMR n=1 | *p*-value |
| --- | --- | --- | --- | --- | --- | --- |
| NK in glomeruli per mm^2^ glomerular area, median (IQR) | 144 (94 to 216) | 133 (68 to 165) | 57 (17 to 116) | 36 (18 to 50) | 48 | <0.001 |
| NK in PTC per mm^2^ cortical area, median (IQR) | 29 (13 to 34) | 28 (13 to 42) | 13 (8 to 17) | 4 (2 to 8) | 3 | <0.001 |
| Abbreviations: MMDx, Molecular Microscope Diagnostic System; EABMR, early-stage antibody mediated rejection; FABMR, fully developed ABMR; LABMR, late-stage ABMR; NK cell, natural killer cell; NR, No rejection; PTC, peritubular capillaries; TCMR, T cell–mediated rejection. Sufficient material for calculation of glomerular and total NK cell counts was available for 84/86 patients. | | | | | | |

# Supplementary Table 4. Number of NK cells in relation to missing self and genotypic variants.

| Parameter |  | Number of missing self types | |  | *p*-value |
| --- | --- | --- | --- | --- | --- |
| Missing self | 0 n=40 | 1 n=29 | 2 n=7 | 3 n=2 |  |
| NK cells in glomeruli per mm^2^ glomerular area | 55 (36 to 144) | 53 (23 to 94) | 46 (43 to 96) | 19 (10 to 29) | 0.406 |
| NK cells in PTC per mm^2^ cortex | 13 (3 to 29) | 11 (5 to 18) | 4 (4 to 12) | 3 (3 to 3) | 0.401 |
| Genes | homozygous low | heterozygous | homozygous high |  |  |
| *FCGR3A* | F/F158 n=28 | F/V158 n=39 | V/V158 n=18 |  |  |
| NK cells in glomeruli per mm^2^ glomerular area | 39 (17 to 92) | 58 (32 to 137) | 57 (46 to 144) |  | 0.122 |
| NK cells in PTC per mm^2^ cortex | 4 (2 to 19) | 13 (4 to 29) | 11 (5 to 18) |  | 0.127 |
| *KLRC2* | del/del n=0 | wt/del n=17 | wt/wt  n=67 |  |  |
| NK cells in glomeruli per mm^2^ glomerular area |  | 48 (18 to 64) | 54 (27 to 144) |  | 0.127 |
| NK cells in PTC per mm^2^ cortex |  | 3 (2 to 11) | 12 (4 to 29) |  | 0.004 |
| *KLRK1* | LNK/LNK n=39 | LNK/HNK n=39 | HNK/HNK n=6 |  |  |
| NK cells in glomeruli per mm^2^ glomerular area | 49 (37 to 108) | 57 (17 to 137) | 74 (41 to 148) |  | 0.748 |
| NK cells in PTC per mm^2^ cortex | 8 (4 to 25) | 11 (3 to 29) | 14 (4 to 26) |  | 0.978 |
| rs9916629 | T/T 46 | T/C n=30 | C/C n=8 |  |  |
| NK cells in glomeruli per mm^2^ glomerular area | 46 (25 to 132) | 66 (33 to 101) | 34 (15 to 123) |  | 0.483 |
| NK cells in PTC per mm^2^ cortex | 6 (3 to 27) | 12 (3 to 22) | 28 (9 to 38) |  | 0.312 |
| NK cells, natural killer cell; PTC, peritubular capillaries. Sufficient material for calculation of glomerular and total NK cell counts was available for 84/86 patients.  Values are median (interquartile range). | | | | | |

# Supplementary Table 5. Estimated eGFR slope in patients stratified to the median number of NK cells in glomeruli and PTC

| Number of NK cells | Slope (95% CI) | *p*-value |
| --- | --- | --- |
| NK cell in glomeruli |  |  |
| NK cells < median | -2.51 (-3.53 to -1.48) | <0.001 |
| NK cells > median | -2.71 (-3.72 to -1.70) | 0.637 |
| Intergroup difference | -0.20 (-1.64 to 1.24) | 0.783 |
| NK cell in PTC |  |  |
| NK cells < median | -2.21 (-3.21 to -1.21) | <0.001 |
| NK cells > median | -3.06 (-4.04 to -2.08) | 0.265 |
| Intergroup difference | -0.84 (-2.24 to 0.56) | 0.244 |

NK cells, natural killer cell; PTC, peritubular capillaries

# Supplementary Figure 1. Bland-Altman plot for the agreement of the histological evaluation of NK cells between the two investigators.


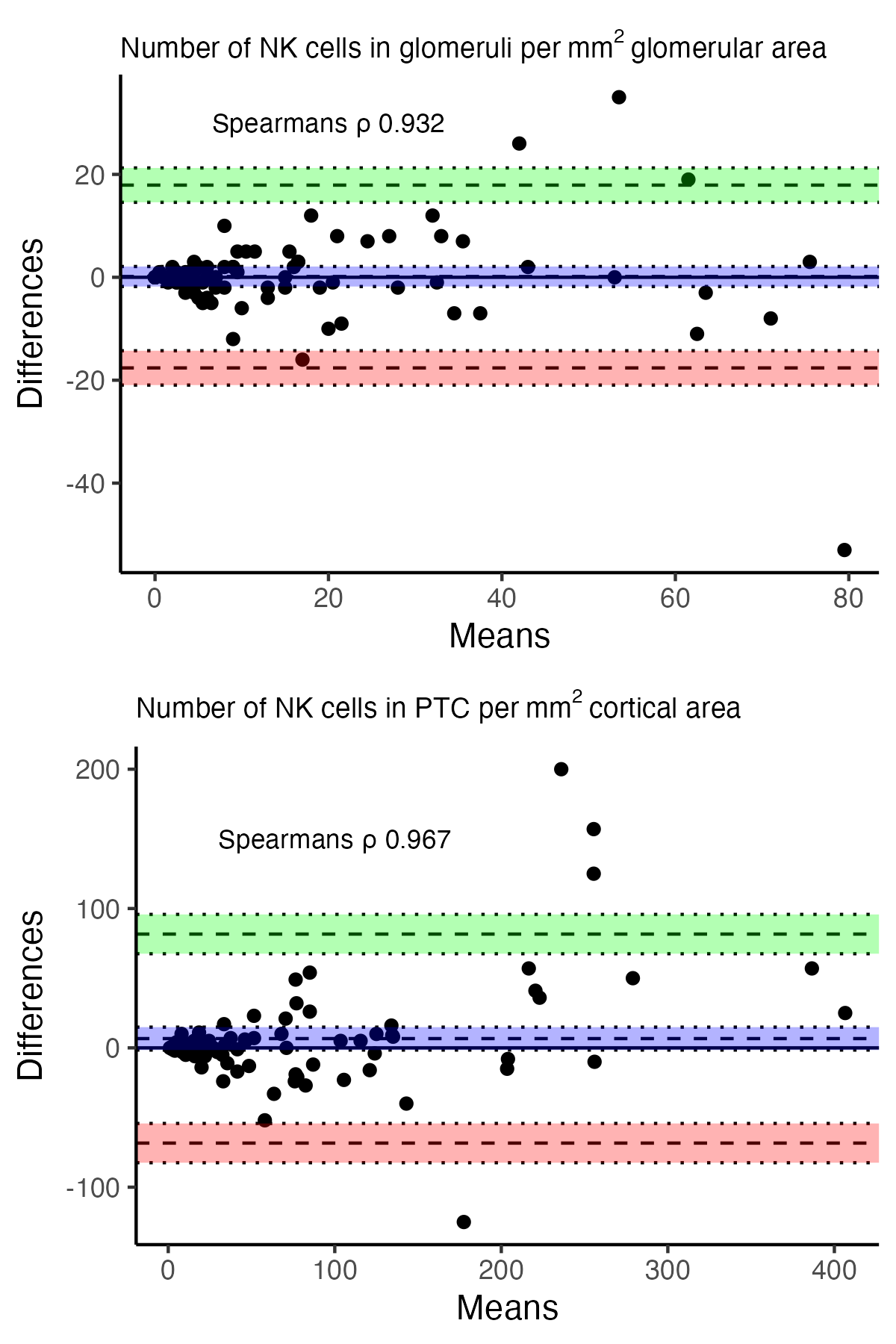


Bland Altman plot displaying the agreement of the calculated natural killer (NK) cells in glomeruli and peritubular capillaries (PTC) per mm^2^ glomerular/cortical area. Shown are the differences between the two measurements on the y axis with the mean of the measurement on the x axis. The blue line represents the mean of the difference, the green and red line represent the mean of the difference ± 1.96 standard deviation. The colored areas represent the confidence interval. In addition, the correlation coefficient using Spearman’s correlation between the two measurements is printed.

# Supplementary Figure 2. Correlation plot between histological markers and the number of natural killer (NK) cells.


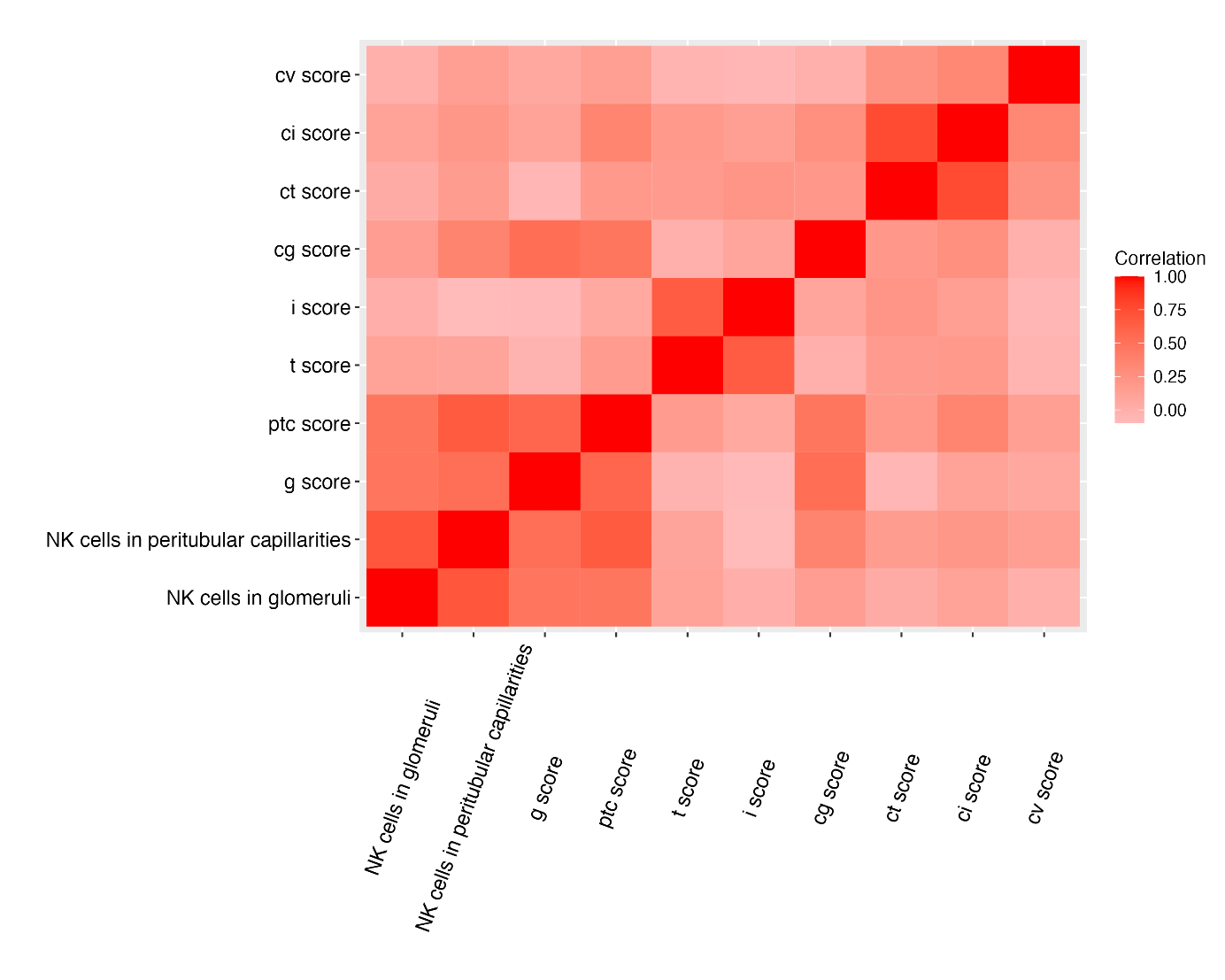


Spearman’s Rank correlation between the Banff single lesions scores and the number of NK cells in glomeruli and in peritubular capillaries per mm^2^ glomerular/cortical area. The different shades of red represent the strength of correlation. Abbreviations: g, glomerulitis; ptc, peritubular capillarities; t, tubulitis; i, interstitial inflammation; cg, double contours; ct tubular atrophy; ci, interstitial fibrosis; cv, vascular fibrous intimal thickening.

# Supplementary Figure 3. Number of natural killer (NK) cells in glomeruli and peritubular capillaries (PTC) and distinct Molecular Microscope Diagnostic System (MMDx) classifiers.


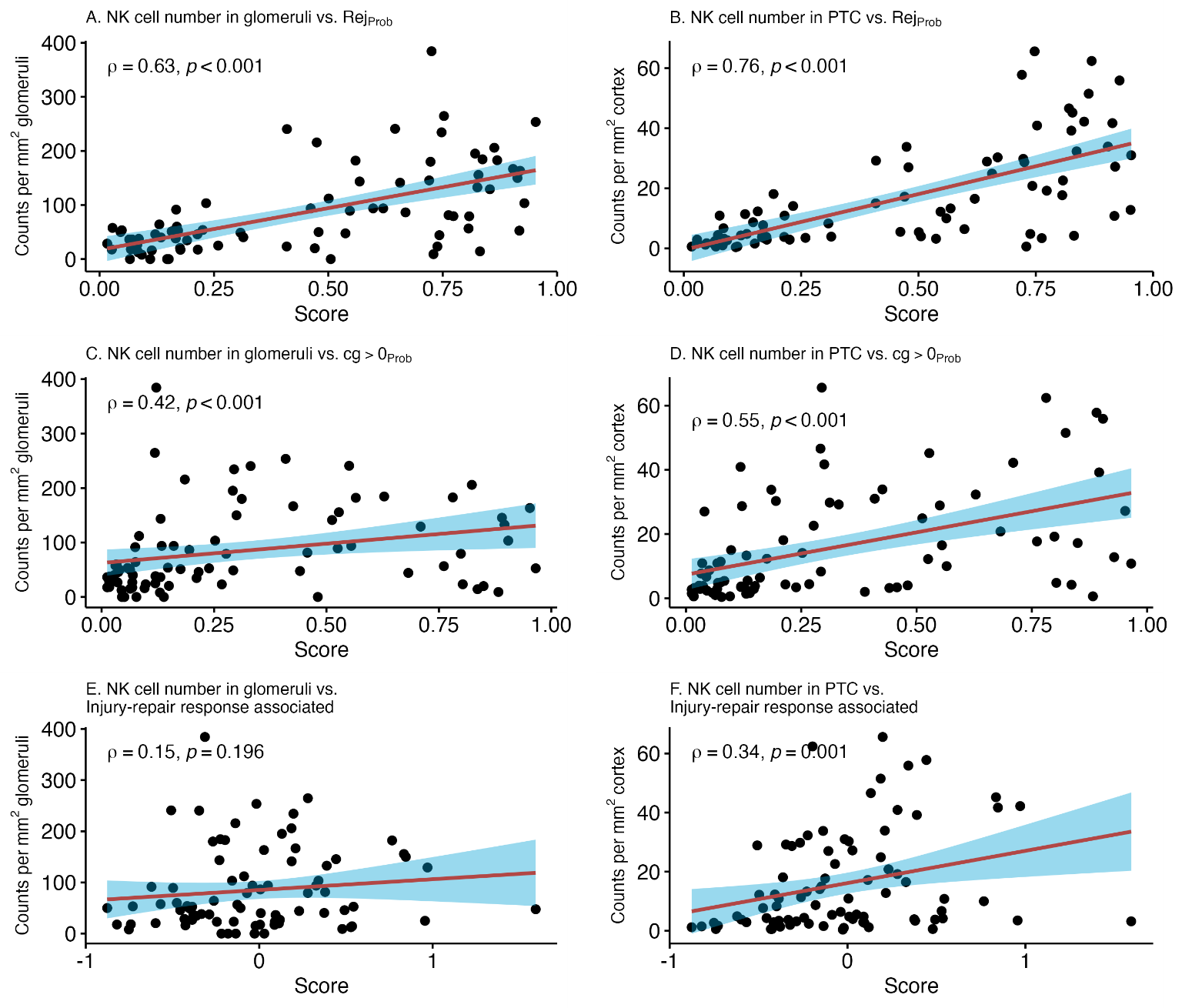


Number of NK cells in glomeruli (left panels) and PTC (right panel) per mm² of glomerular/cortical area and MMDx-derived scores reflecting all rejection’ (Rej_prob_), cg score >0 (cg_prob_) and injury-repair response associated PBT (IRRAT). The red line indicates a linear regression with the 95% confidence interval shown in blue. The Spearman’s correlation coefficient ρ is provided.
